# Inferring ancestry from population genomic data and its applications

**DOI:** 10.3389/fgene.2014.00204

**Published:** 2014-07-03

**Authors:** Badri Padhukasahasram

**Affiliations:** Center for Health Policy and Health Services Research, Henry Ford Health System, Detroit, MIUSA

**Keywords:** global ancestry, local ancestry, hidden Markov models, Bayesian inference, maximum likelihood estimation

## Abstract

Ancestry inference is a frequently encountered problem and has many applications such as forensic analyses, genetic association studies, and personal genomics. The main goal of ancestry inference is to identify an individual’s population of origin based on our knowledge of natural populations. Because both self-reported ancestry in humans or the sampling location of an organism can be inaccurate for this purpose, the use of genetic markers can facilitate accurate and reliable inference of an individual’s ancestral origins. At a higher level, there are two different paradigms in ancestry inference: global ancestry inference which tries to compute the genome-wide average of the population contributions and local ancestry inference which tries to identify the regional ancestry of a genomic segment. In this mini review, I describe the numerous approaches that are currently available for both kinds of ancestry inference from population genomic datasets. I first describe the general ideas underlying such inference methods and their relationship to one another. Then, I describe practical applications in which inference of ancestry has proven useful. Lastly, I discuss challenges and directions for future research work in this area.

## INTRODUCTION

In population genomic analyses, it is often necessary to classify a sample of organisms into different population groups. This can inform us about the evolutionary relationships and migration history of natural populations and help identify an individual’s population of origin. Because both the sampling location of an organism or self-reported ancestry in the case of humans can be uninformative for this purpose, the use of genetic markers can facilitate accurate and reliable ancestry inference by exploiting allele frequency differences across population groups. Recent advances in genomic technologies as well as computing resources have made it possible to accurately infer overall ancestry as well as ancestry at a fine-scale across an individual’s genome. Ancestry estimation is a frequently encountered problem and has been used in a variety of applications such as tracing someone’s geographic origin in forensic investigations, correcting for population stratification in genome-wide association studies and developing personalized approaches to treatment.

There are currently two different paradigms underlying ancestry inference: global ancestry (GA) estimation and local ancestry (LA) estimation. GA inference involves estimating the proportion of ancestry contributed by different populations averaged across the entire genome. Such methods have been applied to study population structure in humans (e.g., Pritchard et al., 2000; Rosenberg et al., 2002; Tang et al., 2005; Price et al., 2006; Lao et al., 2014) as well as in many other species (e.g., Nordborg et al., 2005; Becquet et al., 2007). In contrast, in LA inference, we interpret each chromosome in an individual’s genome as a mosaic of segments that originate from different ancestral populations and the goal is to find the ancestral population of origin at each position. LA inference methods (e.g., Tang et al., 2006; Sankararaman et al., 2008; Maples et al., 2013) have been used mainly to study recently admixed populations such as African Americans and Latinos.

In this mini review, I will describe the various methods that are currently available for efficient and accurate inference of GA and LA from large genomic datasets. I first discuss the general ideas behind the different approaches that are used, their relationship to one another, relative performance in terms of speed and accuracy, advantages, and drawbacks. Then, I will describe many applications in which ancestry inference methods have proven to be useful. Lastly, I will discuss challenges and possible directions for future research in this area.

## METHODS FOR GLOBAL ANCESTRY ESTIMATION

The main goal of GA inference is to estimate the fraction of ancestry contributed by each population as averaged across the entire genome. There are two broad categories of methods available for such inference: model-based approaches and non-parametric approaches.

### MODEL-BASED APPROACHES

Model-based approaches for GA inference attempt to estimate individual ancestry coefficients assuming particular statistical models. For example, the programs STRUCTURE (Pritchard et al., 2000) and ADMIXTURE (Alexander et al., 2009) both model the probability of observed genotypes using ancestry proportions and population allele frequencies assuming Hardy–Weinberg equilibrium and linkage equilibrium among loci. STRUCTURE is based on a Bayesian approach that uses a Markov Chain Monte Carlo algorithm to obtain samples from the posterior distribution. Falush et al. (2003) later extended this method to allow for admixture linkage disequilibrium (LD). InStruct is an extension of STRUCTURE which can jointly infer both population structure and inbreeding rates for organisms that undergo self-fertilization (e.g., plants). The method relaxes the Hardy–Weinberg equilibrium assumption within clusters (Gao et al., 2007). fastSTRUCTURE uses efficient algorithms to infer posteriors underlying the STRUCTURE model employing a variational Bayesian framework. In this framework, posterior inference is posed as an optimization problem (Raj et al., 2014). ADMIXTURE employs the same model (Alexander et al., 2009) as STRUCTURE but uses a maximum likelihood estimation procedure involving high-dimensional optimization algorithms. In particular, this is accomplished through a block relaxation scheme that alternates between updating the ancestry coefficient matrix and population allele frequency matrix. Convergence is accelerated via a novel quasi-Newton method. ADMIXTURE is over an order of magnitude faster than STRUCTURE and produces estimates of similar accuracy (Alexander et al., 2009). The program FRAPPE (Tang et al., 2005) also follows the same likelihood model as STRUCTURE but estimates parameters by maximum likelihood estimation using an Expectation Maximization algorithm. Enforcing strict convergence criteria makes this program computationally burdensome. Therefore, in practice, relaxed convergence criteria are used which makes the results slightly less accurate than ADMIXTURE (Alexander et al., 2009). More recently, Frichot et al., 2014 described fast computational algorithms for ancestry inference that make use of sparse non-negative matrix factorization (sNMF) and least squares optimization to produce estimates of ancestry proportions. In particular, these algorithms produce ancestry estimates that are as accurate as ADMIXTURE but are ∼10–30 times faster in terms of speed. SPA (spatial ancestry analysis) is a recently developed alternate approach that uses explicit probabilistic models for the change in allele frequency in space and uses these to position individuals into two or three dimensional space (Yang et al., 2012).

### NON-PARAMETRIC APPROACHES

Non-parametric methods make use of techniques from multivariate analysis such as cluster analysis and principal component analysis (PCA) to infer structure in the data. The main goal of cluster analysis is to directly find subsets representing the different population groups in the data (e.g., Gao and Starmer, 2007; Lee et al., 2009; Bouaziz et al., 2012). Other techniques such as PCA (Patterson et al., 2006; Price et al., 2006), multidimensional scaling (MDS; Purcell et al., 2007), and principal coordinate analysis seek to construct projections in lower dimensional space that capture a large fraction of the variation in the marker genotypes. The coordinates inferred by such approaches tend to be highly correlated with the geographic locations from where individuals were sampled (Novembre et al., 2008; Wang et al., 2012). EIGENSTRAT (Patterson et al., 2006; Price et al., 2006) is a well-known program that implements PCA.

One of the issues with methods such as STRUCTURE and ADMIXTURE is that they only consider individual markers and not their joint variation patterns. Markers on the same chromosome tend to be inherited together in the absence of recombination. For close markers, at a population level, this results in LD, i.e., non-random associations that reflects shared genealogy and invalidates the independence assumption. For dense polymorphism datasets such as those obtained from sequencing, haplotype based analysis has the potential to leverage this information and provide improved ability to detect population substructure. ChromoPainter and fineSTRUCTURE (Lawson et al., 2012) are recently developed programs that aim to make use of haplotype structure for high quality PCA and population structure inference respectively. The modeling of LD leads to more accurate structure inference but at a cost of significantly higher running times as compared to programs such as PCA and ADMIXTURE.

## METHODS FOR LOCAL ANCESTRY INFERENCE

Additional complexities to ancestry inference can occur when a population arises as a product of two or more divergent populations mixing for a certain period of time (i.e., admixed populations such as African Americans and Latinos). GA inference methods will assign every individual in such populations to more than one group. Admixed genomes are mosaics of segments originating from different ancestral populations and estimating the ancestral proportions and in particular, finding the regional ancestry at each genomic location in such situations is a particularly challenging problem. Most of the methods that have been developed so far take a generative approach to solve this and try to fit an explicit probabilistic model to the data using a hidden Markov model (HMM) or its extensions. Generative approaches for LA inference first try to model the joint dependence of alleles and ancestry and subsequently use “Bayes” rule to estimate the dependence of ancestry on SNP allele configurations.

Early approaches to LA inference based on the STRUCTURE framework (Falush et al., 2003; Hoggart et al., 2004; Patterson et al., 2004) made use of HMMs and did not explicitly model background LD. One limitation of such methods is that they do not fully leverage the information that is available in haplotypes which can potentially be useful for distinguishing closely related populations. In contrast other methods that can explicitly model LD [e.g., SABER: Tang et al., 2006; HAPAA (HMM-based analysis of polymorphisms in admixed ancestries): Sundquist et al., 2008; HAPMIX: Price et al., 2009] are computationally intensive and are able to consider only two ancestral populations at a time. LAMP (local ancestry in admixed populations) is a state of the art algorithm for estimation of LA in recently admixed populations (Sankararaman et al., 2008) that operates on sliding windows of contiguous SNPs and assigns ancestries based on a clustering algorithm. It was shown to be more accurate and significantly faster than STRUCTURE (∼10^4^ times faster) and SABER (∼200 times faster). One of the underlying assumptions is the absence of recombination within windows. WINPOP is a modification of the original LAMP framework that uses a refined model of recombination events and an efficient dynamic programming algorithm to improve LA inference for situations where ancestral populations are closely related (Pasaniuc et al., 2009). PCAdmix (Brisbin, 2010) is a heuristic approach for LA inference. This approach first divides the genome into windows of 10–50 kb width and estimates the probability of origin from particular reference panel populations using PCA. These probabilities are then used as emission probabilities in a HMM to infer ancestry via Viterbi decoding. SupportMix is another recently developed approach for LA inference that trains Support Vector Machines in a sliding window HMM framework (Omberg et al., 2012). ASPCA (ancestry-specific principal components analysis ) is a novel method for inferring the within-continental origin of haplotypes along the genome for admixed populations and was developed recently in the context of reconstructing the history of Caribbean populations (Moreno-Estrada et al., 2013). It involves first inferring LA and then applying PCA to only genomic segments of specific ancestries.

An alternative approach to LA inference, RFMix was developed recently which takes a discriminative approach to this problem (Maples et al., 2013). Such approaches model the dependency of the unobserved variables (i.e., ancestries) directly as a function of the observed variables (i.e., alleles). RFMix makes use of conditional random fields which are based on random forests trained on reference panels. LA inference based on RFMix was shown to be faster and more accurate than many competing approaches such as LAMP (∼33 fold faster) and Support Mix (∼1.7 fold faster). EILA (efficient inference of local ancestry; Yang et al., 2013) is another recently developed statistical method that uses fused quantile regression and a *k*-means classifier to perform LA inference. The method does not assume linkage equilibrium between markers and proposes to use all the genotyped SNPs for greater accuracy. EILA has been shown to be more accurate than programs such as LAMP and HAPMIX when the ancestral distance between populations is large or moderate and is comparable in terms of speed.

### NUMBER OF SOURCE POPULATIONS AND ANCESTRAL MISSPECIFICATION

Many LA inference methods assume that the ancestral source populations as well as their allele frequencies are known and use these as inputs. In practice, such information may either not be available or even if available could be inaccurate (e.g., for Native Americans). Simulation experiments demonstrate that errors in ancestral specification can significantly impact LA prediction accuracy and the drop in accuracy is higher for closely related ancestral populations (Pasaniuc et al., 2009). Thus, choosing accurate ancestral groups is crucial for such scenarios but is less critical when ancestral groups are distant (Pasaniuc et al., 2009). In the absence of ancestral population information, many existing approaches can also utilize the information contained in the admixed samples themselves to estimate LA *de novo* (e.g., Sankararaman et al., 2008; Maples et al., 2013).

Most of these previously mentioned methods have been demonstrated to be highly accurate for the case of two way admixtures such as in African Americans (Seldin et al., 2011). However, the accuracy of such methods declines for more complicated scenarios such as the admixture of three ancestral populations in case of Latinos (European, African, and Native American). The presence of closely related populations in multi-way admixtures (e.g., Europeans and Native Americans) further increases the difficulty of inference. Many existing methods either cannot handle these scenarios or are prone to high error rates making it hard to reliably study LA in such cases. Keeping these issues in mind, several new approaches were developed in the last few years to more effectively handle multi-way admixtures. Johnson et al. (2011) use an extension of SABER to three-way mixtures in a haploid mode to infer virtual genomes. Henn et al. (2012) extended the work of Bryc et al. (2010) to employ PCA and HMMs to estimate ancestries for multi-way admixtures. LAMP-LD and LAMP-HAP (Baran et al., 2012) are extensions of the LAMP algorithm designed for dealing with multi-way admixtures and combine HMMs with an innovative window-based framework to achieve high accuracy estimates in Latinos. Rodriguez et al. (2013) describe a LA inference method ALLOY that utilizes a factorial HMM to capture the process generating maternal and paternal admixed haplotypes, and, inhomogeneous variable length Markov Chains to model the background LD in ancestral populations. ALLOY can handle both recent and ancient admixtures with up to four ancestral populations. Guan (2014) presented a two-layer HMM to detect structure of local haplotypes and demonstrated its utility for LA inference for both two-way and three-way admixture. Lanc-CSV (local ancestry using continent specific variants) is a new method for ultra-fast and accurate inference of LA in very large sequenced cohorts by using continent specific variants in a standard HMM framework (Brown and Pasaniuc, 2014).

## APPLICATIONS OF GLOBAL AND LOCAL ANCESTRY INFERENCE

Ancestry estimation using genomic data has proven to be very useful for many different applications. Importantly, in genetic association studies, ancestry inference can be used to account for the effects of population stratification which is a serious confounding factor and can lead to elevated rates of false positives (Price et al., 2010). In many scenarios, one is interested in the presence of “cryptic” population structure, i.e., structure that is significant and detectable only in genetic terms and not by external features. Estimation of cryptic population structure is also important for DNA fingerprinting to quantify the probability of false matches (Balding and Nichols, 1994, 1995; Foreman et al., 1997; Roeder et al., 1998).

Global ancestry inference is also useful in many evolutionary studies, where we are interested in learning more about the properties of populations and the relationships among them (Cavalli-Sforza et al., 1994). For this purpose, it is useful if we can classify samples into populations. Similarly, given the knowledge of different population groups, one may wish to classify an individual of unknown origin into one of these groups (Davies et al., 1999) or determine if an individual is an immigrant. In the personal genomics space, many private companies now provide ancestry testing products which make use of genome-wide markers from individuals (Royal et al., 2010). This can enable individuals to learn more about the details of their ancestral history and geographical origins. Lastly, GA inference methods have also proven useful for inferring population structure in many non-human species such as maize (*Zea mays*; Pritchard, 2001), chickens (*Gallus gallus domesticus*; Rosenberg et al., 2001), thale cress (*Arabidopsis thaliana*; Nordborg et al., 2005), rice (*Oryza sativa*; McNally et al., 2009), and chimpanzees (*Pan troglodytes*; Becquet et al., 2007).

Like GA inference, LA inference has also found numerous applications. The most important application of LA inference has been to map genes to disease through admixture mapping in populations such as African Americans and Latinos (e.g., Hoggart et al., 2004; Zhu et al., 2004; Reich et al., 2005; Seldin et al., 2011). Other crucial applications have included pharmacogenomics; for example, in a recent study, Native American ancestry was significantly associated with the risk of relapse in children suffering from acute lymphoblastic leukemia (Yang et al., 2011). In addition to these traditional applications, in the more recent years, LA inference methods have also found applications in other settings such as localizing sequences of unknown location from the human reference genome (Genovese et al., 2013), studying recombination rate variation (Hinch et al., 2011; Wegmann et al., 2011), inferring natural selection (Tang et al., 2007; Jin et al., 2012), making demographic inferences (Bryc et al., 2010; Johnson et al., 2011; Kidd et al., 2012), and in joint association and admixture mapping to boost the power to detect disease linked genes and variants (Pasaniuc et al., 2011; Shriner et al., 2011).

## FUTURE RESEARCH AND CHALLENGES IN ANCESTRY INFERENCE

With rapid advances in sequencing technologies, the amount of genomic data available to us has grown massively in the recent years. With the advent of dense variation data from fully sequenced samples of genomes in thousands of individuals (e.g., 1000 genomes project) and advances in haplotype phasing methods, we can anticipate new ancestry inference methods as well as refinement of existing ones to more fully exploit the information available. How to maximally utilize the rich information available in the form of haplotypes in such exhaustive catalogs of variation while developing inference methods that are also computationally efficient and scalable for large sample sizes is an important challenge for the future. For both global and LA methods, there is also scope for improved modeling of background LD between genetic variants that can lead to lower error rates and enhance our ability to detect subtle kinds of population structure. The availability of large genomic datasets also allows us to characterize the geographic locations of individuals with unprecedented detail and more effectively distinguish between closely related population groups. More accurate tools for population structure inference will therefore also lead to more reliable knowledge of the ancestral history for individuals in personal genomics and better facilitate personalized medicine. Similarly, improved methods for LA inference based on such large datasets are also likely to generate more powerful tools for admixture mapping particularly for populations with complex admixture history.

## Conflict of Interest Statement

The author declares that the research was conducted in the absence of any commercial or financial relationships that could be construed as a potential conflict of interest.
